# Rabies in Cats—An Emerging Public Health Issue

**DOI:** 10.3390/v16101635

**Published:** 2024-10-19

**Authors:** Christine Fehlner-Gardiner, Gyanendra Gongal, Tenzin Tenzin, Claude Sabeta, Paola De Benedictis, Silene Manrique Rocha, Alexander Vargas, Natalia Cediel-Becerra, Luis Carlos Gomez, Joanne Maki, Charles E. Rupprecht

**Affiliations:** 1 Ottawa Animal Health Laboratory, Canadian Food Inspection Agency, Ottawa, ON K2J 4S1, Canada; 2 World Health Organization Regional Office for South-East Asia, New Delhi 110 002, India; gongalg@who.int; 3 World Organisation for Animal Health, Sub-Regional Representation for Southern Africa, Gaborone P.O. Box 25662, Botswana; t.tenzin@woah.org; 4 Department of Veterinary Tropical Diseases, University of Pretoria, Pretoria 0110, South Africa; claude.sabeta@up.ac.za; 5 Istituto Zooprofilattico Sperimentale delle Venezie, 35020 Legnaro, Italy; pdebenedictis@izsvenezie.it; 6 Department of Health and Environment Surveillance, Ministry of Health of Brazil, Brasilia 70.719-040, Brazil; silene.rocha@saude.gov.br (S.M.R.); alexander.vargas@saude.gov.br (A.V.); 7 Facultad de Ciencias Agropecuarias, Universidad de La Salle, Bogotá 111131, Colombia; nmcedielb@unisalle.edu.co; 8 Instituto Nacional de Salud, Bogotá 111321, Colombia; lcgomez@ins.gov.co; 9 Boehringer Ingelheim Animal Health USA Inc., Athens, GA 30601, USA; joanne.maki@boehringer-ingelheim.com; 10 College of Forestry, Wildlife and Environment, Auburn University, Auburn, AL 36849, USA; charleserupprechtii@gmail.com; 11 College of Veterinary Medicine, Auburn University, Auburn, AL 36849, USA

**Keywords:** cat, emerging infectious disease, lyssavirus, One Health, rabies, zoonosis

## Abstract

Human rabies cases today are predominantly associated with infection from rabid domestic dogs. Unlike dogs, a common global reservoir species that perpetuates rabies viruses (RABV) within their populations, domestic cats are much less frequently reported or vaccinated. Epidemiologically, cats are important vectors of lyssaviruses but are not viral reservoirs. Typically, cats are incidental hosts only, infected with the predominant lyssavirus in their geographic locale. Human cases associated with rabid cats have occurred in Africa, Asia, Europe and throughout the Americas. As adept, solitary hunters, wild and domestic felids are at risk of lyssavirus infection based upon interactions with infected prey, such as bats, or from transmission by other mesocarnivores, such as rabid dogs, foxes, jackals, raccoons, and skunks. Current veterinary vaccines provide safe and effective immunity in cats against phylogroup I lyssaviruses, such as RABV, but not against divergent lyssaviruses in phylogroups II-IV. With the focus upon the global elimination of canine rabies, the emergence of rabies in cats represents a concerning trend. Clearly, education about the occurrence of rabies in cats needs to be improved, as well as the routine vaccination of cats to reduce the associated risks to public health, agriculture, and conservation biology from a One Health perspective.

## 1. Introduction

The mammalian family Felidae comprises ~38–40 diverse species of obligate, typically solitary, carnivores [1]. They range in size from the ~1.0 kg rusty-spotted cat (*Prionailurus rubiginosus*) to the >300 kg tiger (*Panthera tigris*). The domestic cat (*Felis catus*) has the widest distribution of extant felids and is the only felid species introduced into Australia. Felids are effective vectors of rabies virus (RABV), with salivary excretion several days before illness, like dogs (*Canis lupus familiaris*) [2]. Unlike the crushing injuries from canids, bites from felids result in deeper puncture wounds [3]. Globally, when laboratory-based surveillance is adequate, multiple felid taxa have been confirmed rabid [4,5,6,7,8].

After dogs, cats tend to be a commonly reported domestic rabid animal. For example, between 217 and 538 rabid cats were reported during 1946–1965 in the United States of America (USA), with nine cases of cross-species transmission in humans [9]. Unlike canids, there are no known felid reservoirs of RABV. Rather, they are infected with the predominant lyssavirus in their locality [10,11]. Hence, it is more appropriate to refer to the subject of ‘rabies in cats’, rather than ‘cat rabies’, per se. Such cases may result in a single human fatality or cause hundreds of human exposures, requiring extensive epidemiological investigations and costly administration of human postexposure prophylaxis (PEP) [12,13]. While shown to be susceptible to Australian bat lyssavirus, no rabid cats have been documented yet in Australia [14].

Clearly, rabies in cats has significant repercussions to public health, veterinary medicine, and conservation biology [15]. The objective of this perspective was to provide a global overview of the status of rabies in cats, related to surveillance, prevention, and control, and to address future areas in need of further research and development.

## 2. Materials and Methods

This was not an exhaustive nor conventional review on all elements associated with rabies in cats. Rather, authors searched the recent literature from the past five years, concentrating upon reports focused upon cats and rabies in their respective region. In addition, public databases on rabies surveillance, such as from the Rabies Bulletin of Europe, the Pan American Health Organization SIRVERA report, World Organisation for Animal Health (WOAH) World Animal Health Information System (WAHIS) six-month reports, and the annual surveillance summaries from Canada and the USA, were gleaned for relevant trends for a historical appreciation of any substantive changes in time or space.

## 3. Regional Epidemiological Findings on Surveillance, Prevention, and Control

### 3.1. Asia

#### 3.1.1. Southeast Asia and South Asia

**Figure 1:**
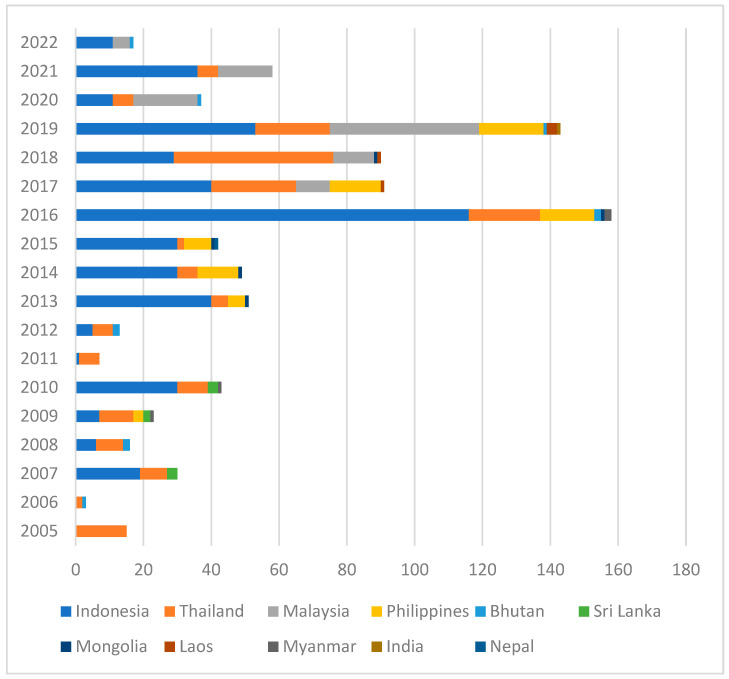
Total number of rabies cases in cats in Southeast and South Asia, reported by year and country to WOAH-WAHIS (January 2005 to June 2022).

Between January 2005 to June 2022, only 11 of 24 countries (Bhutan, India, Indonesia, Laos, Malaysia, Mongolia, Myanmar, Nepal, the Philippines, Sri Lanka, and Thailand) reported 885 cases of rabies in cats (1.7%, 885/52,412 total cases in domestic animals) to WOAH-WAHIS, with Indonesia (*n* = 464) and Thailand (*n* = 204) reporting more cases in cats compared to others during this reporting period (Figure 1 and Figure S1). These data suggest that either cats are less commonly infected with lyssaviruses or that rabies in cats is underreported in most countries, although incidents are likely to be low.

Underreporting is highlighted by the fact that despite some countries not reporting such rabies incidences to WOAH-WAHIS, published studies indicate the occurrence of rabies in cats. For instance, a study conducted from 1998 to 2007 at the Institut Pasteur in Cambodia involved the testing of 1255 animal heads of suspected rabies cases. Dogs constituted 96.7% of the samples, with 49.2% testing positive, whereas only 17.6% of submitted cats were found rabid [16]. In China, studies have documented human rabies deaths resulting from cat exposures. Guo et al. [17] conducted an extensive investigation, analyzing data from 11,902 cases between January 2006 and December 2012 across 30 provinces. Cases due to exposure to domesticated owned/pet cats, domesticated cats from the neighborhood, and free-roaming cats caused 2.7, 0.6, and 0.8% of cases, respectively. Another study covering 15 surveillance sites in 30 provinces from 2005 to 2011 reported 19,221 human cases, primarily linked to injuries from dogs (93.7%), but also cats (4.6%), with the remainder associated with other domestic animals and wildlife [18]. Despite these human cases, cat samples were not tested for rabies at the Chinese National Reference Laboratory for Animal Rabies between 2010 and 2020 [19]. In India, dog-mediated rabies is responsible for most human deaths, while cat exposure accounts for only 2% of human deaths (see Table S1). The details of rabies in cats and human deaths resulting from cat exposure in select Asian countries are presented in Table S1.

#### 3.1.2. Middle East and Caucasus Region

**Figure 2:**
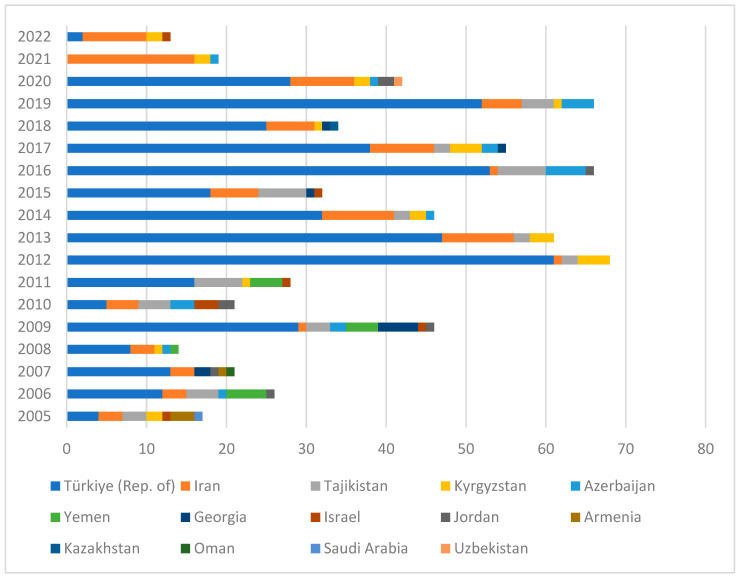
Total number of rabies cases in cats in the Middle East and Central Asia, reported by year and country to WOAH-WAHIS (January 2005 to June 2022).

Only 14 of 24 countries in the Middle East and Central Asia have reported 675 cases of rabies in cats (2.4%, 675/27,716 total cases in domestic animals) to WOAH-WAHIS during the reporting period from January 2005 to June 2022. Among these, Türkiye has reported the highest number of rabies cases in cats, totaling 443 cases, followed by Iran with 94 cases (Figure 2 and Figure S2).

We observed a higher frequency of reported rabies cases in cats from Central Asian countries/Middle Eastern region compared to South and Southeast Asian countries. This observation underscores regional variations in the prevalence and reporting of this disease, which could be attributed to differences in surveillance and monitoring systems.

#### 3.1.3. Control of Rabies in Cats in Asia

In Asia, rabies vaccination campaigns primarily focus on domestic dogs, but cats are also included in the vaccination efforts as part of the rabies elimination program. As up to 99% of all human rabies cases worldwide are transmitted by dogs, with this percentage reaching 96% in Southeast Asia [20], dogs are recognized as the primary cause of rabies-related deaths in humans. Consequently, rabies control measures are directed towards dogs through mass dog vaccination programs, promoting responsible pet ownership, and conducting public awareness education campaigns.

Specific rabies vaccination campaigns targeting cats are rarely conducted in Asia. However, pet owners often bring their cats for vaccination during dog vaccination campaigns. Since domesticated cats also exhibit a behavior pattern of roaming, lyssavirus infections in cats can occur due to spillover infections from dog bites and other RABV-infected animal bites. Therefore, it is crucial to vaccinate domesticated cats not only to prevent the risk of disease transmission from free-roaming cats and dogs but also to minimize the risk of human infection.

### 3.2. Africa, with a Focus on the Republic of South Africa and Zimbabwe

Dog rabies has been present in southern Africa for centuries. The most recent incursion occurred in the 1940s when rabies spread by dogs from Angola and reached South Africa in the 1950s [21]. From the northern regions of South Africa, the disease spread to Mozambique, Eswatini, and then the KwaZulu/Natal Province in 1961 where it has been endemic until today. Dog rabies spilled over into black-backed jackals (*Lupulella mesomelas*) and bat-eared foxes (*Otocyon megalotis*), two wild mesocarnivore species that are capable of maintaining RABV infection cycles independent of dogs in the region [22,23,24,25].

Analysis of historical rabies cases demonstrated that dogs constituted the majority of rabies cases (59.8%), whereas cats were responsible for only 3.2% of the cases in South Africa [26]. Similarly, for the 1928–2000 period, more cases were recorded in dogs than cats (36.9% and 3.7%, respectively) [21,27]. In Zimbabwe, 79 rabies cases in cats were confirmed between 1950 and 1982 [28]. There is generally a lack of information on the ecology of domestic (owned and free-roaming) cats despite the fact that cats are an important vector for rabies. Cats are widespread in urban and rural Zimbabwe and South Africa, and similar to dogs, are closely associated with human habitation [28]. However, the cat population is considerably less than that of dogs. In a survey conducted in the high-density suburbs of Harare (Zimbabwe), only 1.7% of responding households reported owning cats and none were vaccinated against rabies.

During a rabies epizootic (April 1981 and May 1982), six domestic cats from Bulawayo (Zimbabwe) were found to be infected with atypical rabies-related viruses in routine rabies diagnosis [29]. These viruses were later confirmed to be the Mokola virus (MOKV) using the neutralization index in mice. More recently, RABV (*n* = 51) from domestic cats and wildcats (*Felis lybica*) collected between 2010 and 2021 in South Africa were sequenced. After the sequencing and phylogenetic reconstruction, most cats were infected with canine RABV and, to a lesser extent, with viruses from mongooses (e.g., *Cynictis penicillata*). Preliminary analysis showed that one of the cases was due to infection with MOKV [30]. Several cases of this variant, genetically distinct from viruses found in the south of the continent [29,31,32], were previously identified in Nigeria, Cameroon, Ethiopia, Zimbabwe, and the Central African Republic. Spillover infections of Lagos bat lyssavirus (LBV) into domestic cats have been rarely reported (1982 and 2013 in South Africa and 1986 in Zimbabwe) [28,33] since the first detection of LBV in Nigeria in 1956 [21].

Rabies in domestic cats is a potential public health risk given the close association of cats with MOKV and mongoose and canid RABV. Although human rabies cases associated with domestic cat exposures in South Africa are relatively rare, between 1983 and 2018, 13 (2.8%) human rabies cases were linked to domestic cat exposures (infection with mongoose or canid biotypes) [34]. In general, the domestic cat appears to have a close association with smaller wildlife species on which it preys, and this assertion is supported given that the majority of cats were infected with a mongoose RABV variant [28] as well as MOKV. We, therefore, advocate for the continuous vaccination of both domestic dogs and cats against rabies to mitigate the associated public health risks.

### 3.3. A European Perspective

Europe accounts for about 10% of the global human population and has the second highest density worldwide (73/km^2^). Based on the figures available on pet food consumption, more than 100 million cats are estimated to inhabit Europe, with a human to cat ratio of up to 9 [35]. Given the source of the estimate, it likely represents predominantly owned cats but will include other cats that may be cared for/fed without belonging to a specific household. This estimated ratio is also confirmed by cross-sectional surveys in small areas of the continent.

Rabies in Europe is linked to the circulation of RABV in wild non-flying mammals and to at least six non-RABV lyssaviruses associated with specific bat species. The red fox (*Vulpes vulpes*) is the main reservoir of RABV throughout the continent, although other mesocarnivores have also contributed to the final case numbers in wildlife. The raccoon dog (*Nyctereutes procyonoides*) also actively contributed to the maintenance of rabies in the northeastern territories, though its role as a reservoir species is debated [36]. Except for bat lyssaviruses, western countries are currently free of rabies [37]. Official data from the countries contributing to the Rabies Bulletin Europe indicate a resurgence of the infection in previously free areas (such as Poland, Romania, Hungary, and Slovakia) and persistence in endemic countries, such as in Ukraine and Belarus (2016 data) as well as the Russian Federation (2019 data) [38]. The cat displays similar sensitivity as the dog to infection with the fox variant RABV, although it is potentially better able to transmit the infection through salivary excretion [39]. Despite RABV epidemiology in Europe, about 60% of the RABV cases currently reported in the continent have been described in domestic animals [38]. Of them, 40% are found in dogs, 33% in cats, and 23% in cattle [38]. These figures differ from the historical (pre-1990) epidemiology of the infection in the continent, where cats accounted for about 5% of total cases [40], and likely indicate a weak surveillance in wildlife.

According to European Union (EU) regulations (*n*. 576/2013 and 577/2013), vaccination is mandatory for domestic carnivores before movement across and within the EU territories, with the requirement for RABV antibody titration for animals coming from high-risk/endemic countries. However, it must be noted that rabies cases in illegally imported cats are very rare, with one single event of the 22 imported animal RABV cases notified in the EU territories in the last 20 years [41].

As reported to the Rabies Bulletin Europe, human deaths due to locally acquired RABV infection still occur in countries most affected by rabies in red fox [38], with Ukraine counting a constant number of cases over the years [42]. According to official data from Ukraine, Makovska and colleagues [42] noted that the risk of attack on a human due to a rabid cat is about five times higher than that due to a rabid dog, with 22 human cases secondary to a rabid cat exposure and 24 to a dog exposure, out of 63 cases registered from 1996 to 2020.

Considering non-RABV lyssaviruses, cats represent the best candidate to act as an incidental host among domestic animals, due to their hunting behavior coupled with free-roaming lifestyles [43,44]. In this context, predation by cats is the first cause of rescue for bats, with more than 28% of adult bats admitted [44]. Similarly, cat–bat interactions represent approximately 32% of passive lyssavirus surveillance submissions in the United Kingdom [45]. Viral spillover events from bats to cats have been documented in France [46] and more recently in Italy [47], associated with European bat lyssavirus-1 and West Caucasian bat lyssavirus (WCBV), respectively. Clinical signs of these infections varied from furious rabies encephalitis to atypical signs that may have also been linked to concurrent feline immunodeficiency virus infection, such as convulsion, moderate dehydration, and emaciation [46,47].

The occurrence of WCBV infection in a domestic cat has raised renewed public health attention to the circulation of the divergent lyssaviruses for their spillover risks [48]. In this regard, while it is known that rabies vaccines elicit viral neutralizing antibodies against phylogroup I viruses, though in cats it is to a lesser extent than in dogs and humans [45], they do not confer acceptable cross-protection against phylogroup II/III/IV lyssaviruses [49,50]. However, further investigations are needed to unveil the actual public health risk and significance of the circulation of non-RABV lyssaviruses in their natural hosts. Indeed, despite the dogma of their invariable pathogenicity, an increasing body of evidence indicates that each lyssavirus might display different neuroinvasiveness and host spectrum [50,51] with, e.g., WCBV being pathogenic in certain rodents but not in domestic ferrets (*Mustela furo*), and Lleida bat lyssavirus being poorly pathogenic in rodents [52,53].

### 3.4. South America

#### 3.4.1. Brazil

In Brazil, significant strides have been made in addressing public health issues associated with dog-mediated rabies. However, the control of dog-mediated rabies remains crucial, given the current impracticality of controlling rabies in wildlife reservoirs, due to widespread circulation throughout the country. Domestic cats are identified as a high-risk species for RABV transmission to humans. In Brazil, there are an estimated 22.1 million cats (including ~8 million unowned cats), and with 14.1 million households having at least one cat, the cat population potentially exceeds that of dogs [54].

From 2000 to 2023, of 208 recorded human rabies cases in Brazil, 41.3% (86/208) were dog-related, while 51.9% (108/208) were diagnosed with the antigenic variant 3 (AgV3) RABV from the common vampire bat (*Desmodus rotundus*). Among these, 90.7% (98/108) were directly transmitted by bats and 4.6% (5/108) by cats. During the same period, 404 domestic cats tested positive for RABV, with around 10% showing genetic lineages from bats [55].

Over the last decade there has been a noticeable reduction in human rabies cases caused by canine RABV variants. From 2014 to 2023, of the 32 human rabies cases registered in Brazil, only 1 was dog-mediated, 1 was transmitted by marmoset (*Callithrix jacchus*), and 2 by crab-eating foxes (*Cerdocyon thous*). The remaining 28 cases were diagnosed with AgV3. Of these, 78.6% (22/28) were transmitted directly by bats, 17.8% (5/28) by cats, and 3.6% (1/28) by cattle [55].

Human rabies cases caused by cats with AgV3 detection result from novel dynamics in the interaction between bat species and other mammals that are susceptible to infection with RABV [56,57]. This shift may stem from disturbances in ecological balances throughout the country. The successful control of canine rabies in Brazil has led to an obvious change in the epidemiological profile of the disease, with several factors contributing to the evolving epidemiological profile and the rise in human rabies cases from cats and bat RABV variants. Bats adapting to urban areas due to deforestation, fires, increased mobility, and urban expansion may provide closer contact to cats and dogs. Lack of public awareness about rabies vaccination for domestic cats, difficulties in including cats in vaccination campaigns, and insufficient knowledge about human prophylaxis after cat bites are additional contributing factors. The behavioral characteristics of cats directly impact bat predation and are likely to result in increasing rabies case incidence, especially in large urban centers with a high cat population.

The proximity between humans and bats in both urban and rural settings should be viewed within a broader environmental management context. Understanding constant ecological transformations and considering the dynamics of productive factors and processes are crucial. As such, strengthening of cat vaccination campaigns is also imperative.

#### 3.4.2. Colombia

Before the arrival of the Spanish conquistadors, it is likely that RABV was only present in bats in the New World [58]. Rabies was rare or absent among the dogs at the time and canine-associated RABV arrived for the first time during the second half of the 18th century, when European dog breeds were imported during colonization. Control of dog-mediated rabies in Colombia began in the early 1960s, while wildlife rabies control only began in the 1980s. Decentralization of public health services in Colombia in the 1990s created fragmentation of the national control of rabies and other infectious diseases in several departments of the country. Activities such as animal rabies vaccination, rabies surveillance, and animal population control programs for dog-mediated rabies were performed unequally [59].

According to the National Institute of Health [60], in the period from 2004 to 2022, there were 98 cases of animal rabies, of which 72 cases (73.5%) corresponded to the dog RABV genetic lineage, 24 cases (24.5%) corresponded to wildlife rabies (hematophagous and fruit-eating bat RABV genetic lineages), and in 2 cases (2.0%) the lineage could not be identified. Over the last 19 years, the Caribbean region of Colombia has been classified as “high risk”, with the department of Magdalena reporting the highest case incidences and being the only region that has presented cases of both dog-mediated rabies (in 61 dogs and 15 foxes) and wildlife-mediated rabies (in 3 cats and 1 hematophagous bat). In this department, cross-species transmission has been identified with cases resulting from dog–dog; dog–fox–dog; dog–cat; and bat–cat transmission [60].

One of the most striking aspects of the landscape of rabies epidemiology in Colombia is the role of the cat as the main transmitter of RABV to humans in the country. A review of the evolution of rabies cases in cats from 2017 to 2020 demonstrated that in all the cases described, the animal was either unvaccinated or did not have a current vaccination status. Between 2003 and 2012, a peak of human cases (*n* = 14) occurred in rural Colombia and unvaccinated cats were the most frequently reported aggressor. Human patients were mostly children and females, and the exposure primarily corresponded to bite and puncture lacerations in the hands. The RABV AgV3 was the RABV lineage detected in most cases. The cases occurred along the Andean corridor, an epidemiological hotspot for rabies in vampire bats [61].

Cats have increased in number as pets, due to their relative independence and seeming ease of care [62]. This growth in the pet population continued during the period of confinement during the COVID-19 pandemic, which may be explained by the lack of social interaction experienced by many people for more than a year and the resulting desire for pet companionship [63,64]. This increase in the cat population and the known epidemiology of rabies in cats points to a need for the expansion and intensification of cat vaccination campaigns, particularly among rural populations, and should be combined with health promotion campaigns ensuring the cold chain of vaccines in these remote areas. The private sector and non-governmental organizations should be integrated into these efforts. Importantly, surveillance of domestic animal populations should be designed to address the economic and cultural disparities between rural and urban populations to reduce the neglect of remote communities, particularly indigenous populations in Colombia [65].

### 3.5. A North American Perspective

**Figure 3:**
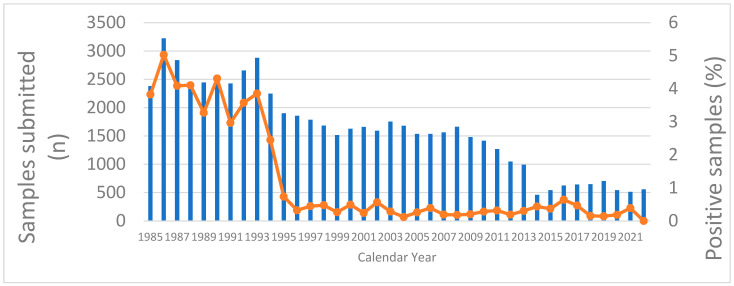
Cats submitted for rabies testing and percent positive samples in Canada, 1985–2022. Blue bars = number of samples submitted for testing; orange dots and lines = percentage of samples that tested positive for rabies. (source: Canadian Food Inspection Agency, unpublished data).

Dog-mediated rabies was eliminated from Canada and the USA during the 1960s and 2007, respectively [66,67]. At present, only wildlife-associated RABV variants are known to circulate in both countries, including viruses associated with Arctic (*Vulpes lagopus*), red (*V. vulpes*), and gray foxes (*Urocyon cinereoargenteus*) and raccoons (*Procyon lotor*), striped skunks (*Mephitis mephitis*), and numerous species of insectivorous bats [15]. Human rabies cases are infrequent in both countries, and those due to cat exposures are exceedingly rare, with only four cases in Canada documented from 1817 to present [68] and 12 in the USA from 1946 to present [9,69,70,71]. Large numbers of suspect rabid cats are submitted for testing each year, exceeding the number of dogs and wildlife reservoirs such as foxes and skunks tested in each country (Figure 3, Table 1). Although the USA tests about 100-fold more suspect animals than Canada, both countries report that approximately 1% of cats submitted test positive compared to ~0.3% of dogs [72]. From 2000 to 2021, there were 3.5 times more rabid cats reported in the USA than dogs (6024 cats and 1697 dogs; mean = 274 cats/yr.; range 216–321/yr.) (Figure S3). The steep decline in the positivity rate for cats in Canada between 1993 and 1996 (Figure 3) is attributable to highly successful oral vaccination programs to control fox rabies in the province of Ontario [73,74]. With the elimination of canine-mediated RABV transmission in Mexico, few cases have been reported in cats (e.g., 11 domestic cats, 1 wild felid, and 1 associated human case in 6 years from 2017 to 2022 [15,72,75,76,77,78]), compared an average of 49 cat cases per year in the 6-year period from 1994 to 1999 [75]. However, 8/11 of the recent cases in domestic cats were found in the State of Yucatan, with 7 cases attributable to an atypical sylvatic RABV variant, and one caused by AgV3, highlighting the increasing importance of spillover from wildlife reservoirs [76].

Although cat case counts are relatively low and there have been few human deaths attributable to exposure to a rabid cat, rabies in cats is a growing public health concern in North America. The cat population is large—owned cats are estimated at 8.5–9.1 mil in Canada and 60–62 mil in the USA, with free-roaming cats estimated at 1.5–4.1 mil and 30–100 mil, respectively [84,85]. Cats are less likely to receive veterinary care than dogs, contributing to population growth, but also importantly, lower vaccination rates. This is in part due to lack of access to veterinary professionals in remote and underserved communities [86,87]. However, even in well-served areas, failure of the public to recognize the value of veterinary care for their cats as well as lack of legislative incentive compelling vaccination of pet cats, are influencing factors. A total of 32 USA states have mandatory cat vaccination, and only 1 out of 13 provinces and territories in Canada requires cat vaccination [88,89]. In New York State, a large percentage of cats submitted for rabies testing were either not vaccinated or not current, while dogs were largely vaccinated [90].

While data from Canada are lacking, in the USA exposures to rabid or potentially rabid cats often results in multiple individuals requiring PEP and cat exposures make up a large percentage of individuals administered PEP [91,92]. The USA Centers for Disease Control and Prevention estimated a cost of over $33 million each year for PEP due to cat exposures [90].

There is ample opportunity for infection of unvaccinated cats, with a large proportion of cat owners in the USA and Canada reporting uncontrolled outdoor access for their cats [93,94]. In both countries, the largest number of rabies cases each year are in bat species [80]. As bat RABV variants are not targets for elimination, the opportunity for infection of non-vaccinated cats will continue, even if some mesocarnivore RABV variants are eliminated. Rabies in cats is of concern not only from the human health standpoint. Two recent examples of displacement of cats incubating raccoon RABV variant rabies into raccoon rabies-free regions in the USA highlight the opportunity for expansion of “hot zones” if the viruses were to move from cats into the naïve wildlife populations [95,96]. As long as rabies exists in wildlife, including bats, vaccination of cats must be promoted and practiced to reduce the risk of human exposures and translocation of RABV variants to new areas, with resultant burdens on public and animal health as well as the economy.

### 3.6. Available Domestic Feline Vaccines

As detailed in the previous sections, rabies in cats is an emerging public health concern in the USA and around the world, but safe, potent, and efficacious commercial vaccines for cats are available. Post-World War II there was a public health need in the USA to prevent human exposures to canine rabies. This need spurred the development of injectable companion animal rabies vaccines. Inactivated RABV vaccines used to vaccinate cats are technologically very similar to those developed for dogs. These products contain inactivated whole RABV particles plus an adjuvant [97]. The adjuvant molecules bind strongly to the RABV antigens enhancing their immunogenicity. The systemic immune response against such rabies vaccines causes a mild inflammatory response. This reaction may result in swelling and tenderness at the vaccination site, lethargy, reduced appetite, and a low-grade fever. Most feline vaccine adverse reactions are mild and transitory [98].

Scientific advancements made in the 1990s resulted in the creation of recombinant viruses expressing heterologous antigens. The first recombinant live viruses evaluated as veterinary vaccines were poxviruses [99]. A canarypox virus vectored recombinant expressing the rabies glycoprotein (ALVAC-vCP65) proved to be safe and efficacious in cats [100,101]. Following subcutaneous injection, ALVAC-cCP65 expresses the rabies glycoprotein in situ, does not produce progeny virus and stimulates a long-lasting systemic immune response against RABV. This vaccine was commercialized and licensed in several countries for use in cats [97,102] (see Table S2 for an annotated bibliography).

In the early 1990s scientists reported an increase in the occurrence of cutaneous tumors in cats identifying these lesions as feline injection-site sarcomas (FISS). Recent studies have shown that FISS continue to be rare (<1 in 10,000 cats) and occur following any injections, including subcutaneous fluid administration, insulin injections, and even with microchip implantation [103,104]. In 1998, the American Association of Feline Practitioners formed a Feline Vaccination Advisory Panel to develop vaccination protocols based on individual risk assessments [105]. This group and others including the American Animal Hospital Association, the European Advisory Board on Cat Diseases, and the World Small Animal Veterinary Association, recognized the value and importance of rabies and other vaccines in the prevention of feline infectious diseases. Recommendations were made to minimize the risk of FISS in cats such as vaccine site mapping and early detection of cutaneous lumps, including biopsies whenever necessary to improve treatment success [106,107,108]. Concern for development of FISS should not supersede the requirement for rabies vaccination of cats.

Safe and effective rabies vaccines are required to address the emerging global zoonotic problem of rabies in cats. To reduce potential human exposure risk, rabies will remain a core vaccine requirement for cats wherever the disease is endemic [106].

## 4. Discussion

Dogs remain the greatest global reservoir of rabies. However, rabid cats have been reported from all inhabited continents, except Australia. It is well understood that there are no feline-specific RABV variants. To date, all documented cases in cats are the result of cross-species transmission with their geographically associated lyssavirus variants. As such, an outbreak with sustained cat-to-cat transmission is unlikely, although not impossible, as other host shift events have been documented, e.g., from *Eptesicus fuscus* (big brown bat) to *Mephitis mephitis* (striped skunks) in Arizona, USA [109]. In addition, cats may be important indicators of the local circulation of non-RABV lyssaviruses, as observed in the Old World. Regardless of the specific viral variant with which it is infected, a rabid cat is capable of transmission onto a tertiary host, including humans, as has been shown in the regional examples above. Documented cases of human rabies cases associated with domestic cat exposures are rare in comparison to canine rabies. However, cases in both cats and humans may be under-reported. A literature review of published studies from Asia revealed documented cases of human rabies exposures due to cat bites and scratches in some countries that were not in official reports (see Section 3.1 and Table S1). This highlights the importance of considering information from multiple sources, including research studies, to gain a comprehensive understanding of rabies occurrences in different regions.

In Asia and Africa, dogs are still the main reservoir of RABV. Dog bites are the primary source of human exposure to RABV. The occurrence of rabies in domestic cats is less commonly reported than in domesticated dogs in both continents. In other parts of the world, notably Europe and the Americas, the importance of dog-mediated rabies is greatly diminished, or even non-existent, due to effective programs for the control and elimination of canine rabies. While successful control of rabies in several mesocarnivore species (e.g., red fox) has been achieved as well (e.g., [73,110,111]), it is unlikely that all bat RABV variants could be eliminated. Cat behaviors, in particular bat predation, the propensity for cat owners to permit unsupervised outdoor access, and the growing population of owned and unowned cats unlikely to be vaccinated against rabies, all increase the likelihood of infection from rabid bats. In South America, predation by vampire bats is also a concern and an emergent means for infection of cats [112,113]. In recent years, there have been increasing reports of human cases in South America transmitted by cats. Such risks posed by cats exhibiting neurologic signs may be largely under appreciated by the public or even health care professionals and appropriate medical care may not be sought nor administered.

A comprehensive understanding of the role of cats in the epidemiology of rabies is rather limited. Further research is needed to provide a clearer picture of wild and domestic felid involvement in the transmission of RABV and other lyssaviruses. Enhanced surveillance and viral characterization should continue as a mechanism to delineate the domestic or sylvatic sources of exposure in cats and to better appreciate the epidemiological importance of emerging lyssaviruses in Eurasia and Africa.

## 5. Conclusions

While they do not serve as primary reservoirs, cats are an important vector and source of RABV exposure to humans and other animals. Where canine RABV perpetuates, cats are typically infected by exposure to rabid dogs. If canine rabies has been eliminated, wild mesocarnivores and bats are important sources of viral exposure for cats. Pure, potent, safe, and efficacious parenteral vaccines exist for application in cats. As with other domestic animals at risk of RABV exposure, all cats should be vaccinated. Rabies in cats will continue while there are unvaccinated, unsupervised cats in rabies enzootic areas. Strengthening surveillance of rabies in cats is crucial not only for understanding the transmission dynamics within the animal populations but also for assessing the potential spillover of RABV from dogs and wildlife to cats. This proactive approach can save lives, both human and feline, and contribute to the overall control, prevention, and elimination of rabies.

## Figures and Tables

**Table 1:** Cat samples submitted for rabies testing in the USA, 2015–2022 [15,72,77,78,79,80,81,82,83].

Year	Submitted (*n*, ×1000)	Positive (*n*)	Positive (% of Tested Samples)
2015	23.1	244	1.1
2016	21.8	257	1.2
2017	21.2	276	1.3
2018	21.8	241	1.1
2019	21.2	245	1.2
2020	16.9	288	1.7
2021	18	216	1.2
2022	17.1	222	1.3

## Data Availability

No archived datasets were analyzed or generated during the study and no new data were created. Specific questions or comments may be sent to the authors ad hoc.

## Supplementary Materials

The following supporting information can be downloaded at: https://www.mdpi.com/article/10.3390/v16101635/s1, Figure S1: Total number of rabies cases in cats in countries in Southeast and South Asia reported to World Organisation for Animal Health-World Animal Health Information System (WOAH-WAHIS) (January 2005–June 2022); Figure S2: Total number of rabies cases in cats in Middle East and Central Asia countries reported to World Organisation for Animal Health-World Animal Health Information System (WOAH-WAHIS) (January 2005–June 2022); Figure S3: Number of reported rabid cats and dogs in the United States per year (2000–2021); Table S1: Overview of rabies cases in cats and human deaths due to cat exposures in select Asian countries; Table S2. Canarypox vectored rabies vaccine literature review. References [16,114,115,116,117,118,119,120,121,122,123,124,125,126,127,128,129,130,131,132,133,134,135,136,137,138,139,140,141,142,143,144,145,146,147,148,149,150,151,152,153,154,155,156,157,158,159,160,161,162,163,164,165,166] are cited in the Supplementary Materials.
