# Supplementary material for: Rabies in Cats—An Emerging Public Health Issue
Source: Viruses. 2024 Oct 19;16(10):1635. doi: 10.3390/v16101635 (PMC11512395; doi:10.3390/v16101635)
Supplement: Supplementary file 1 [file viruses-16-01635-s001.zip › viruses-3229919-supplementary.pdf]

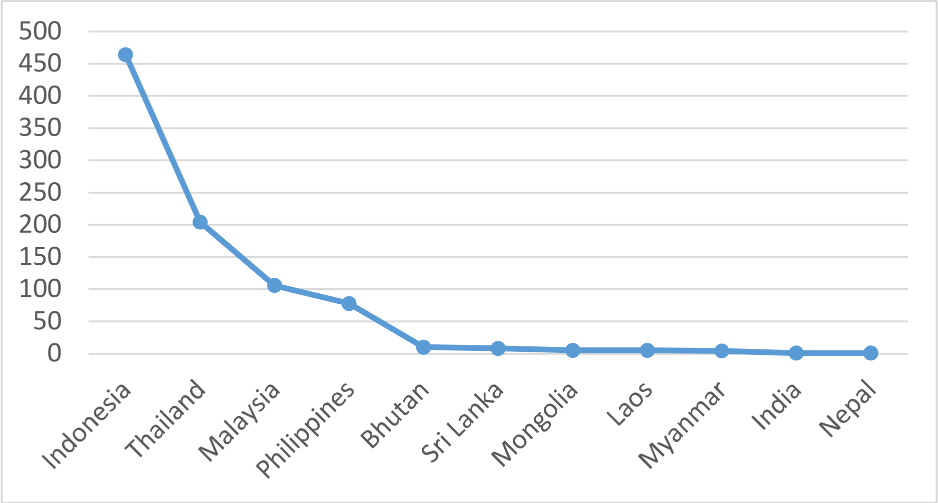

Figure S1: Total number of rabies cases in cats in countries in Southeast and South Asia reported to World Organisation for Animal Health-World Animal Health Information System (WOAH-WAHIS) (January 2005-June 2022)

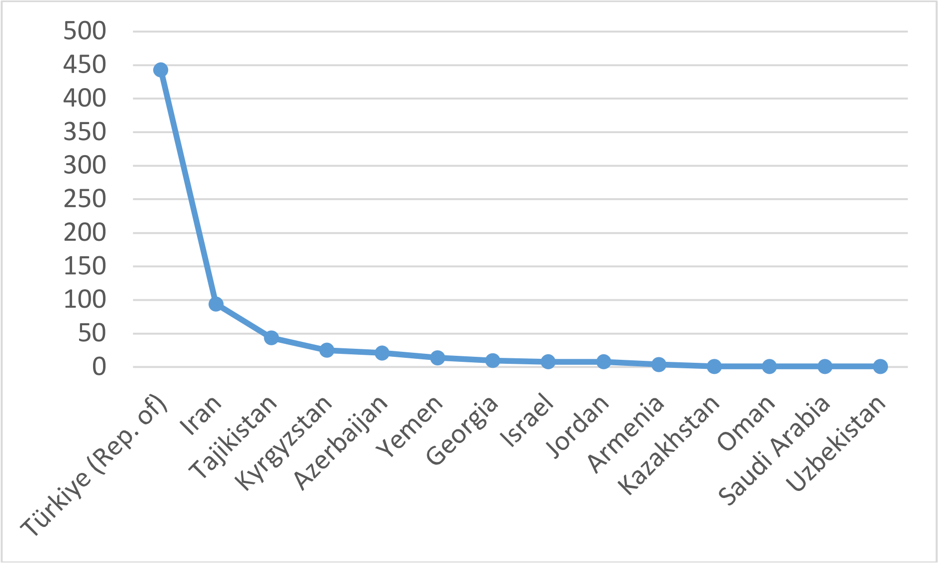

Figure S2: Total number of rabies cases in cats in Middle East and Central Asia countries reported to World Organisation for Animal Health-World Animal Health Information System (WOAH-WAHIS) (January 2005-June 2022)

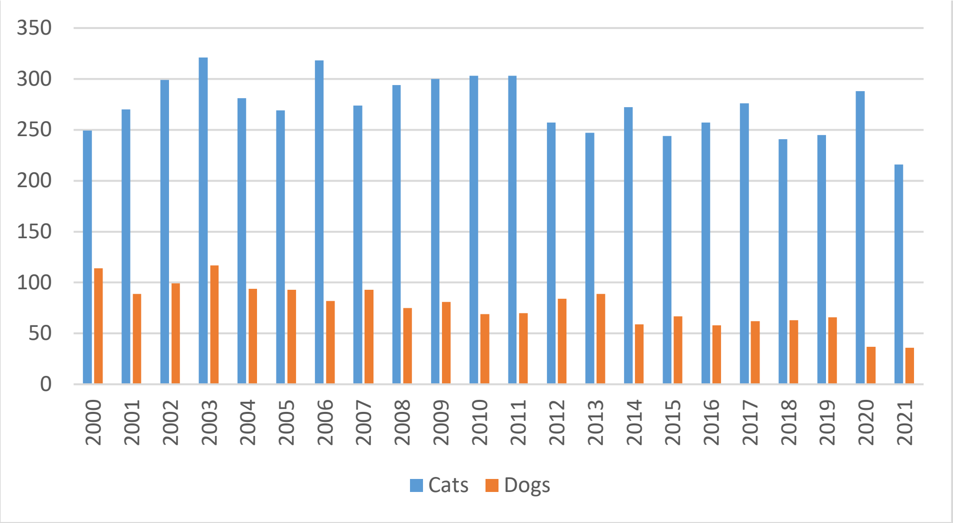

Figure S3: Number of reported rabid cats and dogs in the USA per year (2000-2021) [data sources: 70, 73, 74, 75, 76, 77, 78, 79, 110, 111, 112, 113, 114, 115, 116, 117, 118, 119, 120, 121, 122, 123].

Table S1: Overview of rabies cases in cats and human deaths due to cat exposures in select Asian countries.

Country	Study period and area	Rabies in cats	Human deaths due to cat exposures	References
Afghanistan	-	Unknown	Dog bite is the main source of rabies; no information on cat exposure	[124]
Bangladesh	2013-2015	Presence reported	Of 256 human rabies deaths recorded, 81.6% caused by dog bites and 31 (12.11%) deaths linked to cat exposure	[125]
Bangladesh	2006-2018 IDH Dhaka	Presence reported	Of 1327 human rabies deaths, 380, (90%) caused by dog bites and 24 (6%) deaths linked to cat exposures	[126]
Bhutan	1996-2023	22 (2%) rabies cases in cats reported	19 human rabies deaths reported between 2006 and 2023, all due to dog bites; no human rabies deaths recorded due to cat exposures	[127, 128]
Cambodia	1998-2007	Of 1255 animal heads tested, 211 cat samples tested positive to RABV (17.6% positivity)	63 human deaths recorded at the Institut Pasteur in Cambodia and all due to dog bites; no deaths due to cat exposures recorded	[16]
China	2006-2012 (30 provinces)	present	11,902 human rabies deaths investigated, and cat exposures accounted for 4.1% of deaths	[17]
China	2005-2011 (30 provinces and 15 surveillance sites)	present	19,221 human rabies cases investigated, and cat exposure linked to 4.6% of deaths	[18]
China	-	present	95% of human rabies deaths due to dog bites while cats contributed to around 5% of all cases	[129, 130]

India	2005	Cat cases reported in WOAH-WAHIS	17,137 human rabies deaths estimated/annually; 2% of deaths attributed to cat exposures	[131]
India	2005	present	140 rabies suspect deaths examined through verbal autopsy, and 1 death noted due to cat exposure	[132]
Indonesia	-	Cat cases reported in WOAH-WAHIS	Dog is the main cause of rabies; no information on cat exposures	[133, 134, 135]
Lao PDR	-	Cat cases reported in WOAH-WAHIS	Dog is the main source of rabies with a report of 8,528 dog bites reported annually: no information on cat exposures	[136]
Malaysia	July 2017-Sep 2023 (Sarawak Province)	Brain tissue samples from 847 dogs and 78 cats confirmed positive to RABV (Jul 2015-Dec 2018)	72 human rabies cases with 65 fatalities documented; deaths due to cat exposure also documented	[137, 138, 139]
Mongolia	1996-2005	Cat cases reported in WOAH-WAHIS	Human rabies cases are due to dog bites and wildlife exposures; no information on cat exposures	[140, 141]
Myanmar	-	Cat cases reported in WOAH-WAHIS	Dog bites responsible for 0.2 deaths per 100,000 population annually: no information on cat exposure deaths	[142]
Nepal	-	Cat cases reported in WOAH-WAHIS	Human deaths due to cat exposures not reported	[143, 144, 145, 146]
Pakistan	2009-2019	present	129 human rabies deaths recorded at Indus Hospital and Health Network in Karachi and the Jinnah Postgraduate Medical Center with 1 death due to cat bite reported	[147]
Philippines	2006-2015	Cat cases reported in WOAH-WAHIS	575 human rabies deaths documented, and cats contributed to 3.7% (n = 16) of cases	[148]
Philippines	1987-2005 (Metro Manila)	present	49 (2.9%) human rabies deaths attributed to cat exposures	[149]
Philippines	2020-2022 (Oriental Mindoro)	-	31.5% (3564/11,316) of cat bites in humans recorded, but none resulted in rabies-related deaths	[150]
Philippines	2018-2019 Albay Province	-	9073 (685/100,000 persons) dog bites reported in 13 months; no information on cat exposures	[151]
Sri Lanka	2010-2015	490/2335 (21%) of cat samples tested positive to RABV	Cat exposures accounted for 3% of human cases	[152]
Sri Lanka	2005-2014	661/3273 (21%) cat samples tested positive to RABV	Observed limited cat exposures	[153]
Sri Lanka	2015	607 animal rabies cases, including 471 dogs (77.8%) and 104 cats (17.1%)	Dog bites responsible for around 97%, and cat bites 1.8%, of human rabies deaths	[154]

Sri Lanka	2005-2020	1082/5228 (20.6%) of cat samples tested positive to RABV	No information cat exposures in human	[155]
Thailand	1980-2019	Cat cases reported in WOAH-WAHIS	95% of human rabies deaths due to dog bites	[156]
Thailand	2015 (Eastern Thailand)	present	Dog bites responsible for 77.8% (4824/6204) of human rabies suspect exposures; cats contributing to 19% (1181/6204)	[157]
Thailand	1994-2008, (14 provinces in Southern Thailand)	52 of 331 (16%) cat samples tested positive to RABV	Cat exposures in human not reported	[158]
Thailand	2013-2020 Whole country	156 samples (3.4%) from cats tested positive to RABV	Cat exposures in human not reported	[159]
Vietnam	2012-2018 (south)	present	2 out of 67 human rabies deaths were attributed to cat rabies exposures	[160]

Abbreviations: RABV (rabies virus); WOAH-WAHIS (World Organisation for Animal Health - World Animal Health Information System)

Table S2. Canarypox vectored rabies vaccine literature review.

Reference	Title	Study Design	Results	Comments
[93]	Three-year duration of immunity in cats vaccinated with a canarypox-vectored recombinant RABV vaccine	Duration of Immunity (DOI) and Rabies Efficacy trial according to European Pharmacopoeia; Two SQ doses in combination with other feline antigens given 393 days apart. Controls (11) and vaccinates (30) challenged 3 years (Day 1384) later using fox rabies variant.	Primary vaccination resulted in seroconversion of all vaccinates with a peak 2-8 wk. post-vax. Rabies titers remained stable or slightly declined until booster. Revaccination resulted in strong anamnestic response. All vaccinates survived challenge and rabies confirmed in 10/11 controls	Cats receiving rabies vaccine not in combination with other antigens had higher mean rabies antibody titers that cats receiving rabies vaccine combined with other feline antigens (p = 0.021). Extending DOI to 3 years reduces frequency of required boosters and is another way to reduce the potential for adverse events.
[96]	Efficacy studies on a canary-pox rabies recombinant virus	Efficacy of fowl pox (7.5 $TCID_{50}$/dose) and canarypox rabies (6.5 $TCID_{50}$) in mice by foot pad inoculation and intracranial rabies challenge; 12 dogs	The fowl pox and canarypox constructs protected mice at PD_{50} = 6.17 and 4.18, respectively. Dogs and cats receiving 5.0 or 7.0 logs of	Investigating one inoculation of non-replicating avian virus vectors expressing the rabies glycoprotein induced virus neutralizing antibodies in mice, cats and dogs that were protective

		and 11 cats vaccinated SQ with either 3.0, 5.0, or 7.0 log_{10} $TCID_{50}$/ml and challenged with RABV 26 days after vaccination.	virus produced antibodies and survived challenge while 0/4 dogs and 1/4 cats receiving 3.0 logs survived challenge.	against challenge. This study demonstrates proper antigen presentation by an avian virus to a mammalian immune system resulting in virus neutralizing antibodies and cell mediated responses that were protective against challenge.
[97]	Biological and immunogenic properties of canarypox-rabies recombinant, ALVAC-RG (vCP65) in non-avian species	In vitro and in vivo assessment of the canarypox-based (ALVAC) recombinant vector system. Safety studies in rabbits, guinea pigs and mice by a variety of routes; canaries by dermal application, and non-human primates.	Expression of the rabies glycoprotein insert detected in 6 human cell lines without active virus replication. Pox lesions detected in canaries as expected which resolved by Day 21. Other species produced antibodies without adverse events.	This report contains details of biological and immunological properties of ALVAC vector in a variety of species without evidence of virus replication in human cell lines in vitro and a variety of animal species in vivo.
[161]	Human safety and immunogenicity of a canarypox-rabies glycoprotein recombinant vaccine: an alternative poxvirus vector system	Adult volunteers received IM injections of ALVAC-RG on Day 0, 28, and 180 while cohorts received the standard human diploid cell vaccine (HDCV rabies vaccine on the same schedule.	Human immunogenicity against rabies was demonstrated in volunteers receiving $10^{4.5}$ or $10^{5.5}$ $TCID_{50}$/dose by injection without vector virus interference for booster dose	ALVAC-RG (canarypox rabies) was considered safe in humans and induced antibody and cellular responses to RABV. The recombinant vaccine was immunogenic in humans, but HDCV was superior in generating strong antibody titers compared to ALVAC-RG.
[95]	Applications of pox virus vectors to vaccination: An update	A historical review of recombinant poxvirus vectors expressing heterologous pathogen antigens	Describes human and veterinary recombinant poxvirus vaccines including canarypox rabies results in dogs and cats	For reasons not understood, the canarypox vector is 100 times more efficient than a comparable fowlpox vector vaccine in non-avian species by injection. Future application of pox-virus vectors for novel vaccines is discussed
[98]	Development and registration of recombinant veterinary vaccines. The example of the	A review of the development and registration of ALVAC as vaccine platform for companion animals	As a ubiquitous vector without replication in mammals, the platform was well documented for	Pox virus vectors were accepted first into veterinary medicine and are used in multiple species. In vitro and in vivo tests are performed to evaluate gene

	canarypox vector platform	and horses in the EU and USA.	safety and risk assessments were performed for use in Europe and the USA	transfer and safety in target and non-target species. Due to these additional tests live recombinant vectored vaccines are better characterized that classical attenuated vaccines.
[162]	Immunization with canarypox virus expressing the rabies glycoprotein	Safety and immunogenicity of ALVAC-R SQ injection a three different dose levels in 25 humans and compared to HDCV vaccine. See [161].	Clinical reaction scores after 5 days post-vax were acceptable and inflammation detected in highest dose groups. Antibodies to rabies and canarypox were detected. HDCV could boost ALVAC and ALVAC could boost HDCV.	The vaccines was well tolerated in humans and antibodies demonstrated immunologic response to rabies insert and canarypox vector. There was a dose response effect and both vaccines could boost the other vaccine. The safety of this vaccine was judged sufficient for use in humans. The utility of a non-replicating recombinant vaccine platform for use in humans was discussed.

Abbreviations: DOI (Duration of Immunity); SQ (sub-cutaneous); $TCID_{50}$ (50% tissue-culture infectious dose); IM (intramuscular); PD_{50} (50% protective dose); HDCV (human diploid cell vaccine); RABV (rabies virus); EU (European Union); USA (United States of America).
